# Supplementary material for: High-resolution proteomics unveils salivary gland disruption and saliva-hemolymph protein exchange in Plasmodium-infected mosquitoes
Source: Nat Commun. 2025 Nov 20;16:10205. doi: 10.1038/s41467-025-64837-6 (PMC12635079; doi:10.1038/s41467-025-64837-6)
Supplement: Supplementary file 1 — Supplementary information [file 41467_2025_64837_MOESM1_ESM.pdf]

Supplementary Information

Methods

Mosquito tissue expansion microscopy (MoTissU-ExM)

MoTissU-ExM was performed as previously described (1). Briefly, *P. berghei*-infected salivary glands were dissected and fixed in 4% v/v paraformaldehyde. Fixed salivary glands were anchored in formaldehyde/acrylamide solution before being transferred to a 12 mm round coverslip. On the coverslip, salivary glands underwent gelation in monomer solution (sodium acrylate, TEMED, ammonium persulfate, acrylamide, bis-acrylamide in PBS). Following gelation, coverslips were incubated in denaturation buffer (sodium dodecyl sulfate, Tris, sodium chloride in PBS) at 95 ˚C for 90 minutes. After denaturation, gels were expanded in deionised water before shrinking in 1 x PBS and staining with primary antibodies diluted in 3% BSA-PBS. Gels stained with primary antibody were washed with PBS-Tween 20 before incubation in secondary antibodies plus fluorescent dyes (NHS Ester and SYTOX Deep Red). Following secondary antibody incubation, gels were re-expanded in deionized water and imaged using a Zeiss LSM900 with Airyscan2 (Oberkochen, Germany).

All MoTissU-ExM images in this study were captured using a 40x C-apochromat autocorr M27 objective lens (1.2 NA). Tiled images were made using the lowest possible zoom for this objective (0.45x), with stitching performed using the ZEN Blue (Version 3.8) stitching function. The NHS Ester channel was used as the reference, with a 10% overlap, and 'fuse tiles' and 'correct shading' enabled.

Fourier transformations of saliva patterns

Using ImageJ (FIJI, Version 2.14.0), the NHS Ester Alexa Fluor 405 (protein density) channel was separated, and square regions of interest containing saliva were defined. Ten areas of interest came from secretory cavities containing sporozoites, while 10 regions of interest came from secretory cavities lacking sporozoites. Each region of interest underwent Fourier transformation by using the function: Process > FFT > FFT. The presence of structure in the Fourier transformed image was interpreted as representing saliva that was more heterogenous, while the absence of structure in the Fourier transformed image was interpreted as representing saliva that was more homogenous.

Sporozoite Collection and Immunofluorescence Assay (IFA)

Sporozoites were collected either from the hemolymph or the salivary glands. Hemolymph sporozoites were harvested by perfusion from infected mosquitoes 17 days post-infection, following the procedure detailed in the Hemolymph Collection section. Hemolymph was pooled from 40 mosquitoes (10 µL each), then centrifuged at 5,000 × g for 5 minutes at 4 °C to collect the cell suspension. After discarding the supernatant, the pellet was washed three times by adding an equal volume of PBS and centrifuging again, taking care not to disturb the pellet. The sporozoites were resuspended in 100 µL PBS before the IFA. Salivary gland sporozoites were harvested from infected mosquitoes 21 days post-infection; for this 40 pairs of salivary glands were macerated in 100 µl of PBS.

For immobilization, sporozoites were centrifuged onto poly-L-lysine–coated 15 mm round coverslips (Sigma-Aldrich) at 1,000 × g for 5 minutes, then allowed to adhere for 15 minutes at room temperature. Samples were fixed for 20 minutes in PBS containing 4% (w/v) paraformaldehyde and 0.01% (v/v) glutaraldehyde (Sigma-Aldrich), followed by three PBS washes. Fixed samples were blocked for 1 hour in a blocking buffer consisting of 2% (w/v) BSA and 1% (w/v) goat serum in PBS. Slides were then incubated for at least 2 hours with primary antibodies diluted in blocking buffer. These included rabbit monospecific antibodies against Lp, Transferrin 1, TEP15, and AAPP each at 1 µg/mL. For PPO6, a rabbit anti-PPO6 serum—kindly provided by Dr. Ryan Smith (University) — was used at 1:1000. Anti-ClipA14 and Anti-vitellogenin were used at 1:400 (Boster Bio). The mouse monoclonal anti-CSP (clone 3D11) was also applied at 1:1000. For negative controls, primary antibodies were omitted, except for CSP. After washing, slides were re-blocked for 1 hour and incubated for at least 2 hours with secondary antibodies (goat anti-rabbit or anti-mouse) conjugated to Alexa Fluor 488 (green) or Alexa Fluor 594 (red) (1:2000 dilution; Thermo Fisher Scientific). Nuclei were counterstained with Hoechst (2 µM in PBS) for 10 minutes. The Coverslips were mounted with ProLong Gold Antifade Reagent (Invitrogen) and imaged using a Leica TCS SP8 confocal microscope equipped with a HC PL APO CS2 63×/1.4 NA oil-immersion objective, a 405 nm diode laser, and a white light laser (WLL) (Leica Microsystems, Wetzlar, Germany). Optical z-sections with 0.1 µm spacing were acquired using the LAS X software. Image processing—cropping, resizing, and brightness adjustments—was performed using Adobe Photoshop (Adobe Systems, San Jose, CA).

1. B. Liffner, T. L. A. e. Silva, J. Vega-Rodriguez, S. Absalon, Mosquito Tissue Ultrastructure-Expansion Microscopy (MoTissU-ExM) enables ultrastructural and anatomical analysis of malaria parasites and their mosquito. *BMC Methods* **1**, 13 (2024).

Figures

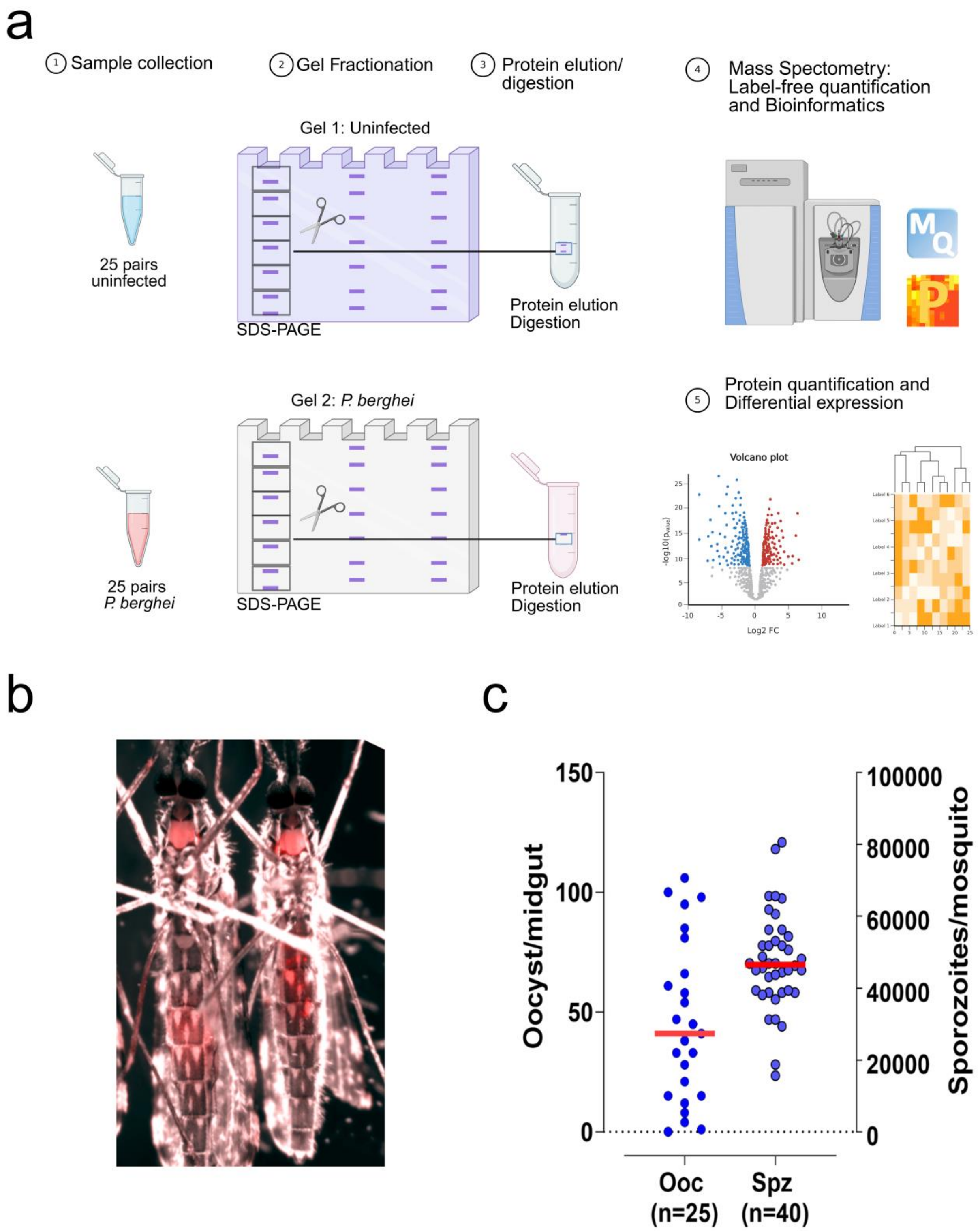
a
1 Sample collection
2 Gel Fractionation
3 Protein elution/ digestion
4 Mass Spectometry: Label-free quantification and Bioinformatics
Gel 1: Uninfected
25 pairs uninfected
SDS-PAGE
Protein elution Digestion
Gel 2: P. berghei
25 pairs P. berghei
SDS-PAGE
Protein elution Digestion
5 Protein quantification and Differential expression
Volcano plot
Log2 FC
b
c
Oocyst/midgut
Sporozoites/mosquito
Ooc (n=25)
Spz (n=40)

Supplementary Figure 1: Experimental design and sorting *P.berghei* mCherry-infected *An. gambiae* mosquitoes. a. Experimental design: Mosquito salivary glands were dissected 21 days post-infection with *Plasmodium berghei*, using three independent infections. Each biological replicate consisted of three pools of 25 pairs of salivary glands from either infected or uninfected mosquitoes. Samples were separated by SDS-PAGE, and six gel fractions were collected. Proteins were extracted and digested with trypsin, and then analyzed by label-free quantification. Created in BioRender. Silva, T. (2025) https://BioRender.com/ne12hsn. **b.** Infected mosquitoes were sorted by fluorescence (mCherry as exemplified in the figure or GFP) with a Leica M205 fluorescence stereo microscope FCA coupled with a DFC 7000 G5 camera. **c.** Distribution of oocysts and sporozoites in a representative proteome experiment.

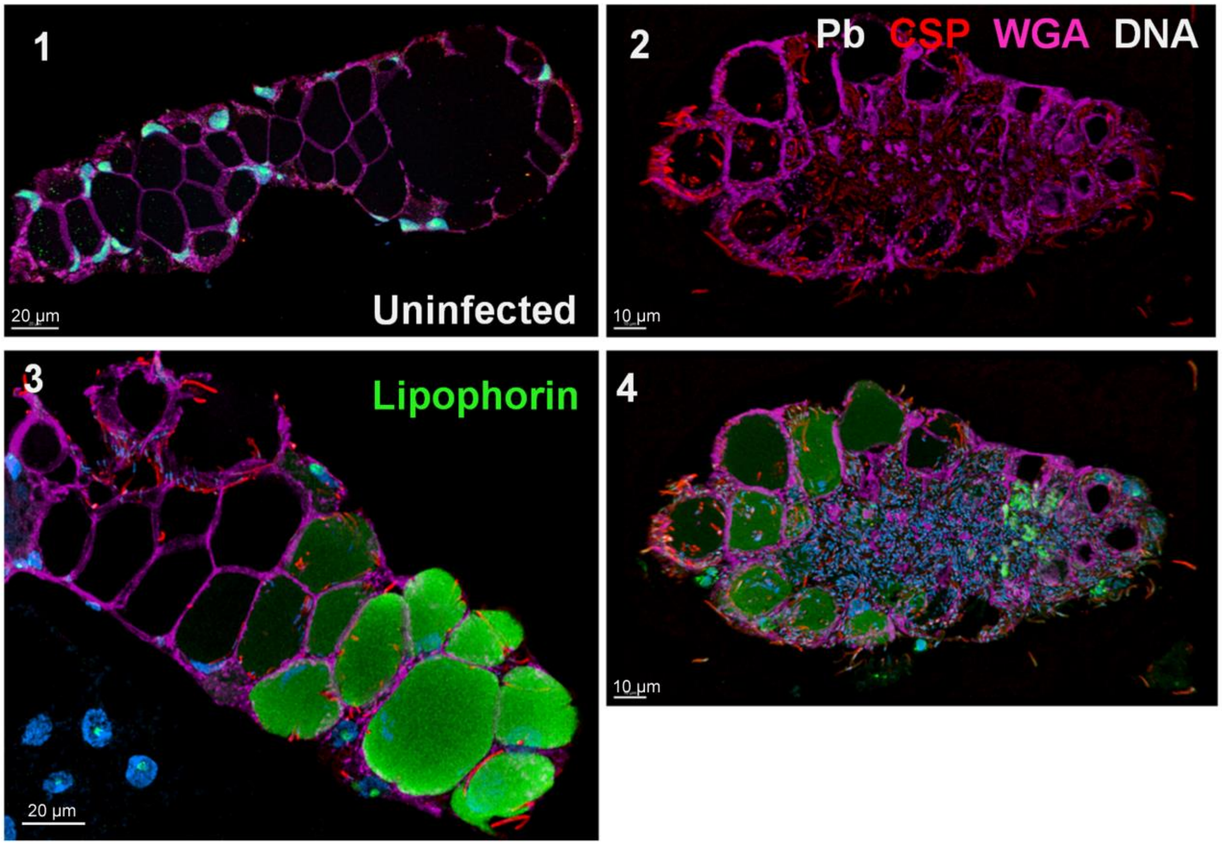

Supplementary Figure 2: Immunofluorescence assay of lipophorin accumulation in infected salivary glands. Staining: Lipophorin green, CSP red, DNA blue, WGA magenta. 1 uninfected, 2-4 *P. berghei* infected salivary glands. Infected salivary glands show increased accumulation of lipophorin.

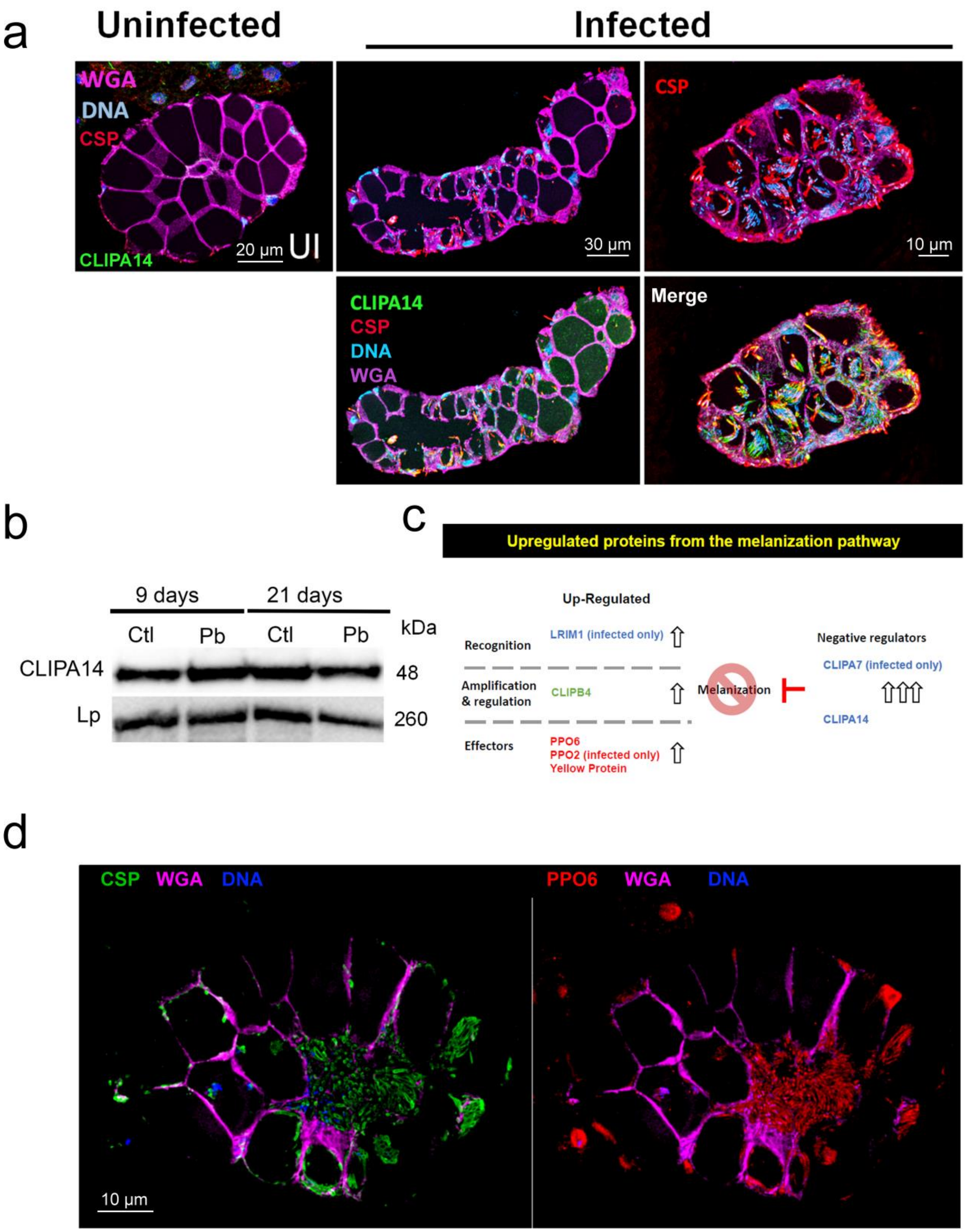

Supplementary Figure 3: Histological immunofluorescence assay of uninfected and *P. berghei* infected salivary glands. a CLIPA14 green, CS red, WGA magenta, DNA blue. **b** Western Blot Analysis of CLIPA14 Expression:

Western blot showing CLIPA14 expression in uninfected (UI) and *P. berghei*-infected (Pb) hemolymph at 9 and 21 days post-infection. At 9 days, no invasion of salivary glands is detected, whereas at 21 days, the salivary glands are heavily infected. After apyrase staining (Fig. 5c), the membrane was stripped and reprobed for CLIPA14. Lipophorin was used as a loading control. **c** Schematic summarizing the upregulated proteins from the melanization pathway. White arrows represent upregulation. **d** PPO6 red, CS green, WGA magenta, DNA blue.

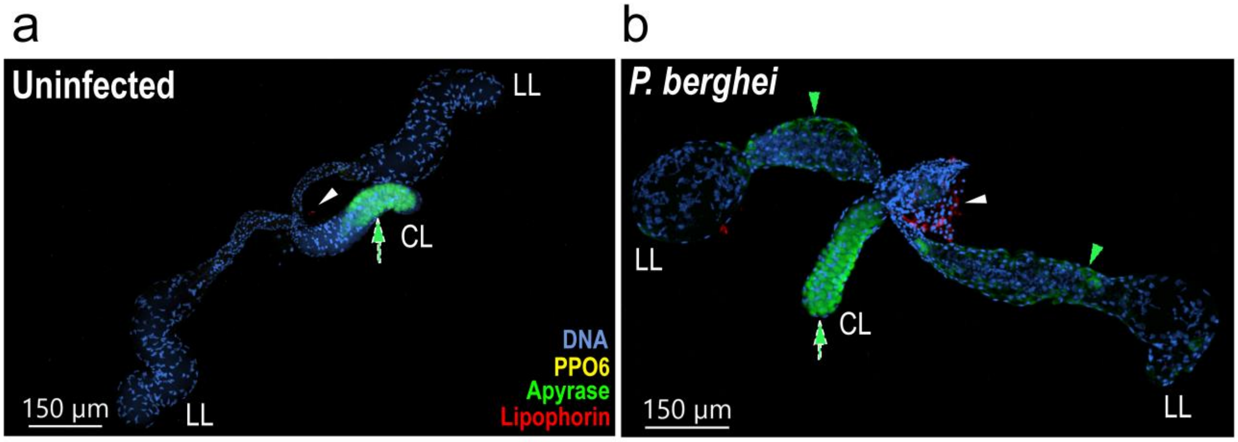

Supplementary Figure 4. Additional examples of the transcriptional activity in uSGs (U) and iSGs (Pb). RNA in situ hybridization in (**a**) uSGs and (**b**) iSGs. Nuclei in blue, PPO6 in yellow, Apyrase in green, and Lipophorin in red. (LL) lateral lobe and (CL) central lobe. White arrows indicate fat body cells associated with SGs, green arrows with white dashed border indicate apyrase mRNA in salivary cavities, green arrows indicate apyrase mRNA in lateral lobes of *P. berghei*-infected salivary glands.

a **Most abundant hemolymph proteins from *P. berghei*-infected mosquitoes 19 days post-infection. Bolded proteins enriched in iSGs.**

Protein IDs	Description
AGAP004977-PA	**Prophenoloxidase 6**
AGAP001826-PA	**Lipophorin**
AGAP006258-PA	Prophenoloxidase 2
AGAP011369-PA	**Gelsolin**
AGAP000376-PA	Transferrin
AGAP011788-PA	**CLIPA14: CLIP-domain serine protease**
AGAP003250-PA	**CLIPB4: CLIP-domain serine protease**
AGAP010968-PA	CLIPA9: CLIP-domain serine protease
AGAP003095-PA	**Yellow protein**
AGAP011789-PA	**CLIPA6: CLIP-domain serine protease**
AGAP008060-PA	**IDGF2: imaginal disc growth factor 2**
AGAP008364-PG	**TEP15: thioester-containing protein 15**
AGAP011792-PA	**CLIPA7: CLIP-domain serine protease**
AGAP003139-PA	Serine protease inhibitor (serpin) 9
AGAP008061-PA	**IDGF4: imaginal disc growth factor 4**
AGAP012616-PA	Prophenoloxidase 5
AGAP004976.P46	Prophenoloxidase 8
AGAP004978-PA	Prophenoloxidase 9
AGAP010658-PA	Hexamerin
AGAP001377-PA	**Serine protease inhibitor (serpin) 11**
AGAP002925-PC	**Poly(U)-specific endoribonuclease**
AGAP004455-PA	GNBPB1: 3-glucan binding protein
AGAP002465-PA	Ferritin heavy chain
AGAP001662-PA	Disintegrin metalloproteinases with thrombospondin repeats
AGAP029749-PB	Trypsin-like serine protease
AGAP002032-PA	Lipoprotein
AGAP007455-PA	LRIM10: Leucine-rich immune protein (Short)
AGAP008364-PD	**TEP15: Thioester-containing protein 15**
AGAP009670-PC	SRPN4: Serine protease inhibitor (serpin) 4
AGAP006278-PA	D7 protein

b

SGs proteome: Proteins up-regulated in iSGs. Abundant hemolymph proteins are bolded

Protein IDs	Description	Fold change
AGAP001569-PB	Myosin Alkali Light Chain 1	17.4854256
AGAP010935-PA	Porphobilinogen Synthase	14.1053903
**AGAP003250-PA**	**CLIPB4**	12.7574957
AGAP001023-PF	Myofilin Variant B	10.6730819
AGAP002350-PB	Troponin T, Fast Skeletal Muscle	10.4584351
**AGAP002925-PC**	**Poly(U)-Specific Endoribonuclease**	9.76303695
**AGAP011788-PA**	**CLIPA14**	6.87116899
AGAP011369-PA	Gelsolin	6.5154338
**AGAP000376-PA**	**Transferrin 1**	4.82313294
AGAP012401-PA	AGM1	3.83590822
AGAP012115-PA	Ca^{2+}-Transporting ATPase	3.50907114
AGAP001826-PA	**Lipophorin**	3.48664891
**AGAP008060-PA**	**Imaginal Disc Growth Factor 2**	3.09660848
AGAP005712-PC	Phenylalanine-4-Hydroxylase	2.87706371
**AGAP004977-PA**	**PPO6**	2.44134122
AGAP006103-PA	AGAP006103-PA	2.27812436
**AGAP008061-PA**	**IDGF4: Imaginal Disc Growth Factor 4**	2.22152879
**AGAP008364-PA**	**TEP15**	2.16172823
AGAP011842-PA	Signal Peptidase Complex Subunit 2	2.09807517
AGAP004960-PA	Prosalpha3: 26S Proteasome Alpha 3 Subunit	1.93335732
AGAP001271-PA	Pre-mRNA Cleavage Complex 2 Protein (Pcf11)	1.91235929
AGAP002335-PB	Nucleolysin TIA-1/TIAR	1.89223474
AGAP001313-PA	Muscular Protein 20	1.85127917
AGAP008774-PA	Cytochrome C Oxidase Assembly Protein	1.65646615

SGs proteome: Proteins unique to iSGs. Abundant hemolymph proteins are bolded

Protein IDs	Description
AGAP005625-PA	SCRASP1: Class A Scavenger Receptor
AGAP003865-PA	gamma-tubulin complex component 3
**AGAP011789-PA**	**CLIPA6**
**AGAP011792-PA**	**CLIPA7**
AGAP007249-PB	Flightin: protein flightin
AGAP001905-PB	zinc finger RNA-binding protein

C **Hemolymph Proteome of Infected Mosquitoes 19 Days Post-Infection: Salivary Proteins Detected Exclusively in Infected Samples. Bolded proteins indicate those with spectral counts present in both replicates**

			P. berghei		Uninfected	
Protein ID	Description	Unique peptides	MS/MS count	MS/MS count	MS/MS count	MS/MS count
**AGAP011026-PA**	Apyrase	14	2	18	0	0
**AGAP006421-PA**	Antigen 5 Related Protein 1	10	3	23	0	0
AGAP008282-PA	D7r2	3	1	4	0	0
AGAP009917-PA	SGS4	68	0	108	0	0
AGAP009918-PA	SGS5	57	0	69	0	0
AGAP008279-PA	D7L2	5	0	7	0	0
AGAP008278-PA	D7L1	4	0	4	0	0
AGAP008281-PA	D7r4	4	0	4	0	0
AGAP009974-PA	Anopheline Antiplatelet Protein	2	0	2	0	0
AGAP008284-PA	D7r1	2	0	2	0	0
AGAP008283-PA	D7r3	2	0	2	0	0

Supplementary Figure 5. Most abundant proteins from proteome analysis of hemolymph, salivary glands, and saliva. **a** List of the most abundant secreted proteins detected by proteomic analysis of hemolymph from *P. berghei*-infected mosquitoes 19 days post-infection. The proteins are ranked from the most abundant to the least. Bolded names are proteins found enriched in infected salivary glands. **b** Proteomic analysis of SGs from uninfected and *P. berghei*-infected mosquitoes. The table lists proteins upregulated or enriched in infected samples and their respective fold changes. Bolded are putative hemolymph proteins enriched in infected salivary glands. The upper table shows proteins found in both uninfected and infected samples, while the lower table lists proteins detected exclusively in infected samples. Proteins abundant in the mosquito hemolymph are bolded. **c** Saliva proteins detected exclusively in the hemolymph of *P. berghei*-infected mosquitoes.

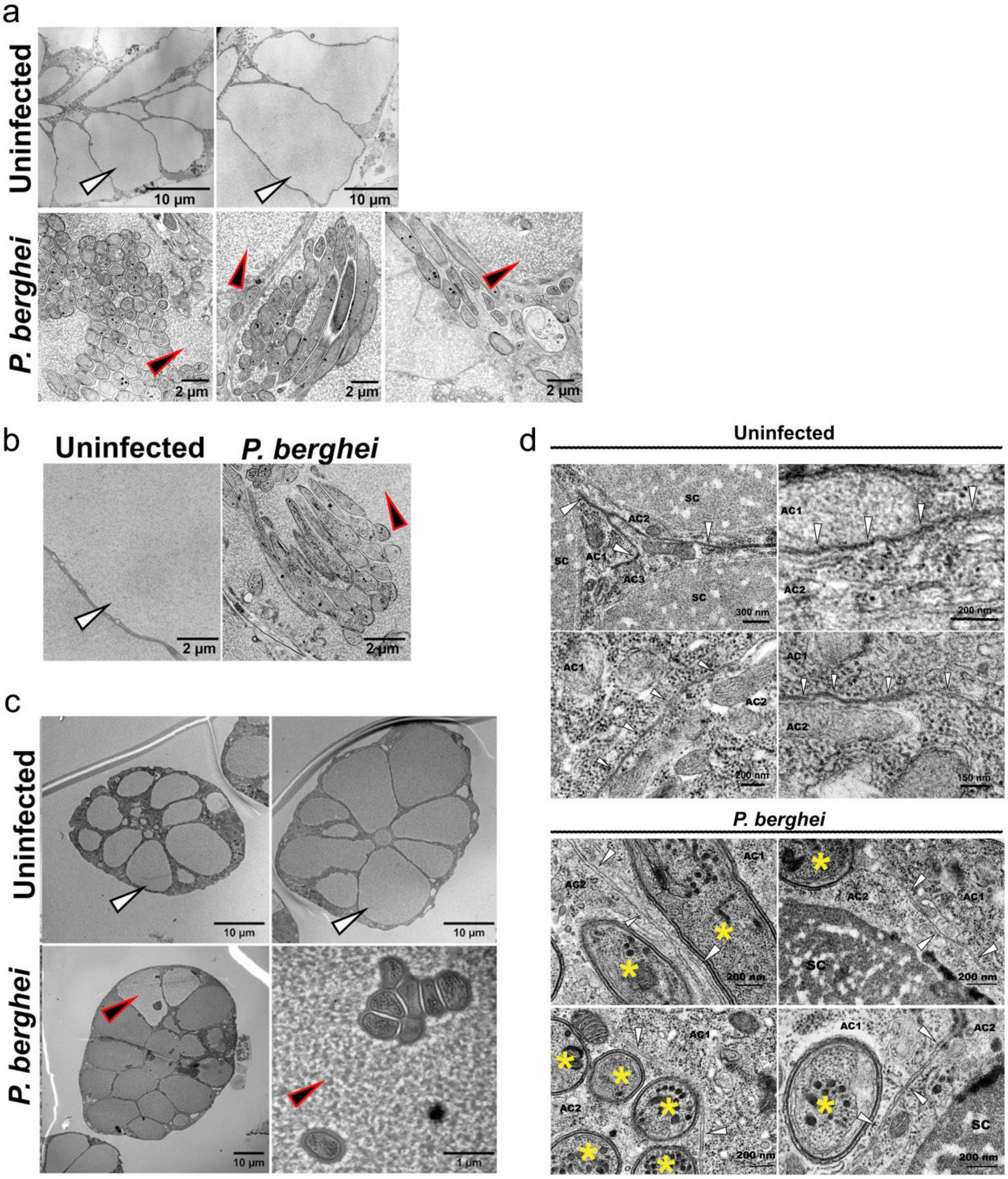

Supplementary Figure 6: *Plasmodium berghei* infection of salivary glands changes the texture of saliva within the secretory cavities. a Uninfected and *P. berghei* ANKA 2.34-infected SGs from *Anopheles stephensi*. **b** A closer view at the same scale of uninfected and *P. berghei*-infected *An. stephensi* SGs. Images in panels **a** and **b** were generated in Dr. Isabelle Coppens' lab at Johns Hopkins University. **c** *An. stephensi* SGs infected with *P. berghei* ANKA 2.34. The arrow

points to an infected cavity with distinct granularity in the saliva matrix. The electrolucent and granular aspects of the saliva are more evident in the bottom right. These images were generated in Dr. Friedrich Frischknecht's lab at Heidelberg University Medical School. **d** Intercellular-junction morphology. Multiple examples of intercellular junctions: in the uninfected glands, acinar cell membranes abut tightly, and the extracellular cleft is absent. In the *P. berghei*-infected glands, the opposite membranes disengage, forming a distinct extracellular space in between the membranes. Acinar cells (AC) and secretory cavity (SC). Sporozoites are marked with asterisks.

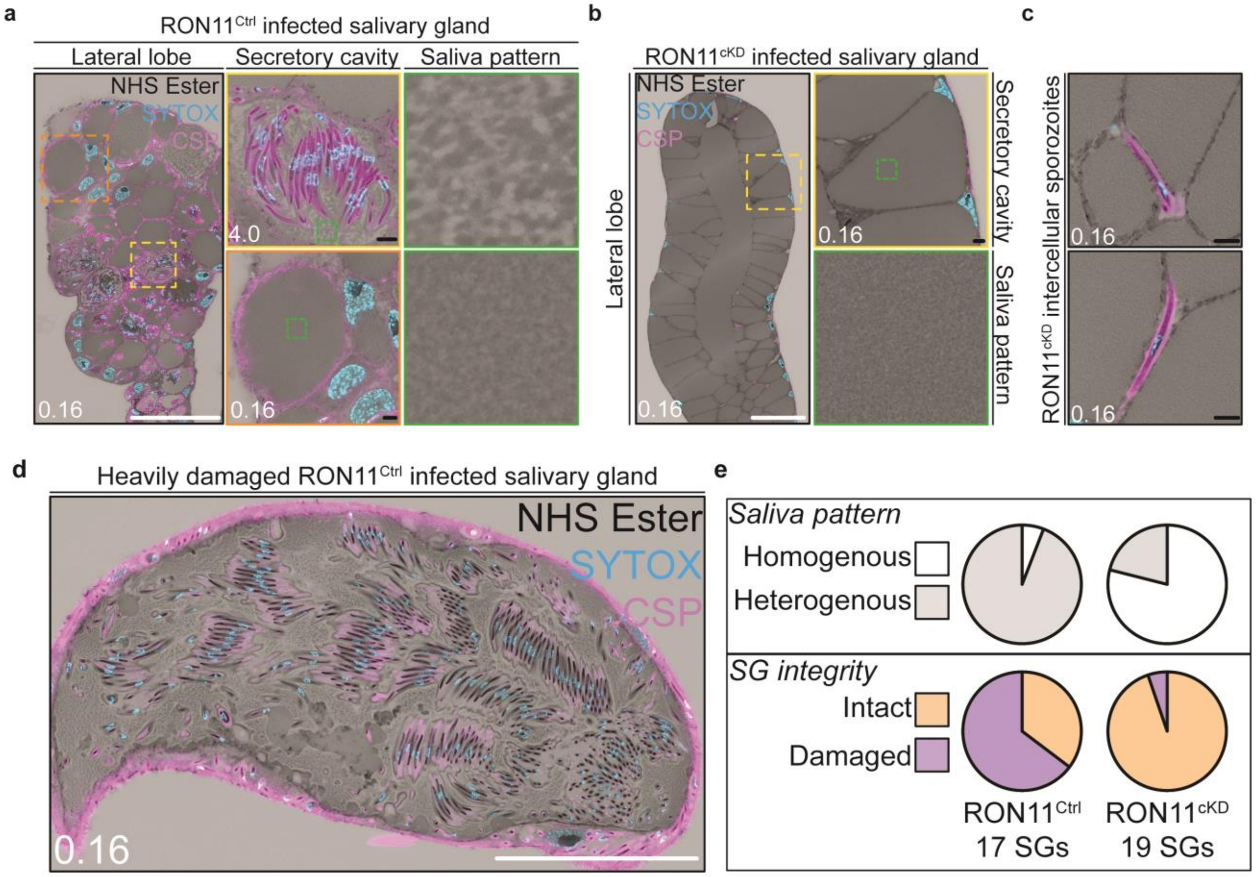

Supplementary Figure 7. Expansion microscopy of iSGs labelled with NHS Ester (the protein density dye, greyscale), SYTOX Deep Red (DNA, cyan), and anti-CSP antibody (sporozoite surface, magenta). **a** Wild-type (RON11Ctl) glands. Insets: uninfected cavities (orange), infected cavities (yellow), and luminal salivary content (green). **b** RON11 conditional knock-down (RON11cKD) glands; yellow arrowheads indicate sporozoites lodged in the intercellular space between adjacent epithelial cells. **c** High magnification images showing intercellular sporozoites (magenta). **d** Representative image of a damaged gland showing the loss of epithelial cell projections that normally delineate each cavity. **e** Quantification of phenotypes. Upper panel: salivary-gland “complexity measured by index” calculated from the power spectrum of the Fourier transformation. Lower panel: proportion of intact versus damaged glands. White scale bars = 200 µm, black scale bars = 10 µm.

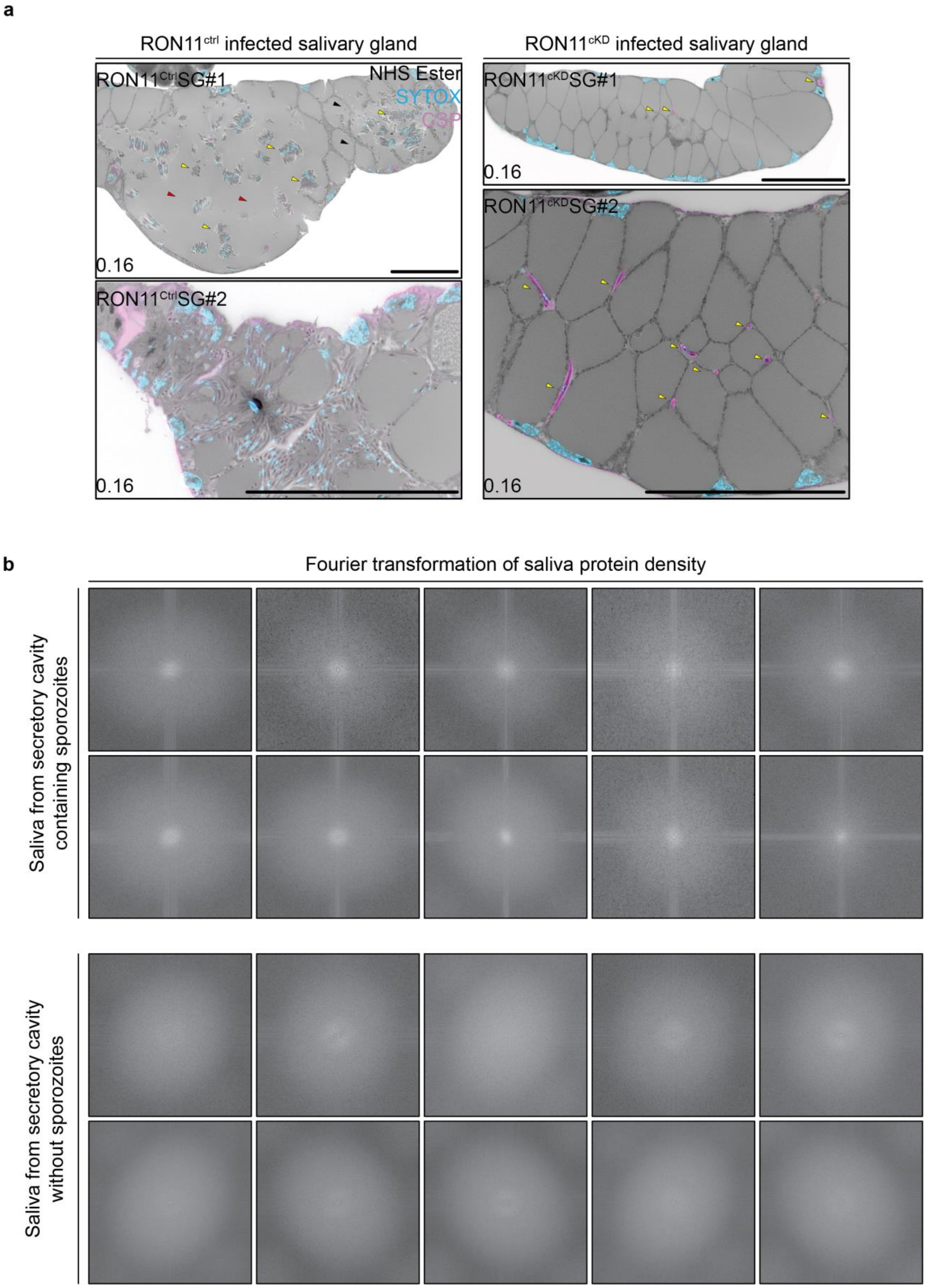
a
RON11ctrl infected salivary gland
RON11cKD infected salivary gland
RON11CtrlSG#1
NHS Ester
SYTOX
CSP
0.16
RON11CtrlSG#2
0.16
RON11cKDSG#1
0.16
RON11cKDSG#2
0.16
b
Fourier transformation of saliva protein density
Saliva from secretory cavity containing sporozoites
Saliva from secretory cavity without sporozoites

Supplementary Figure 8. Expansion microscopy of NHS Ester labeled iSGs. **a** Left: two representative examples of $RON11^{Ctl}$ iSGs. Note areas where the salivary cavities lost cellular delineation (red arrowheads) in contrast with areas with well-delimited cavities (black arrowheads). Right: two representative examples of $RON11^{cKD}$ iSGs. Multiple parasites can be found in the intercellular space (yellow arrowhead). Note the intact aspect of the salivary glands. **b** Two-dimensional Fourier power spectra of NHS-Ester–labeled salivary content from cavities that harbor sporozoites (ten representative glands shown in the upper panel) or that are uninfected (lower panel). Scale bars, 200 µm.

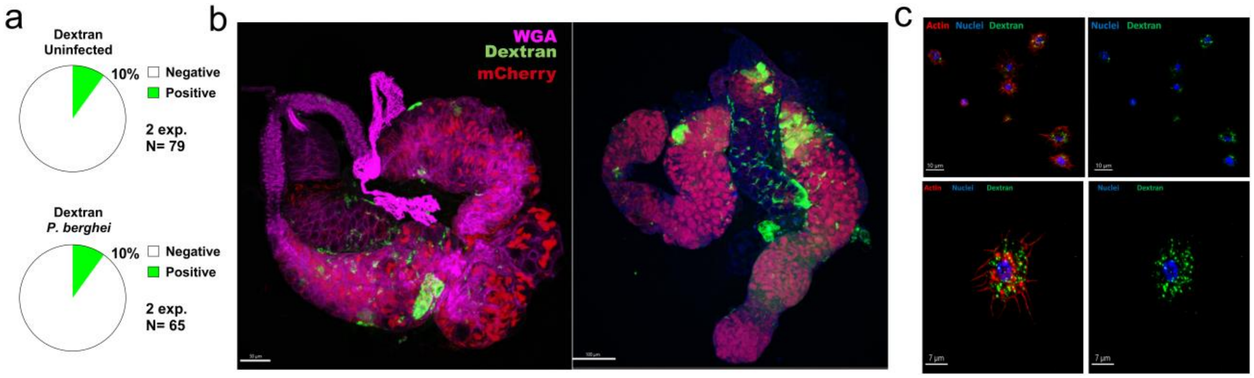

Supplementary Figure 9. Changes in epithelial integrity. a Pie charts of dextran diffusion quantification 9 days pos infection from two independent experiments. **b** Dextran diffusion assay: different patterns of fluorescent dextran (10 kDa) diffusion in iSGs 21 days post-infection. Images display green fluorescent dextran, red mCherry parasites, and magenta WGA staining. **c** Hemocytes uptake dextran injected into mosquitoes. Fluorescent dextran was injected into the mosquito hemocoel, and hemocytes were subsequently collected by perfusing the mosquito hemolymph. Hemocytes are shown with actin (phalloidin) in red, dextran in green, and nuclei (Hoechst) in blue.

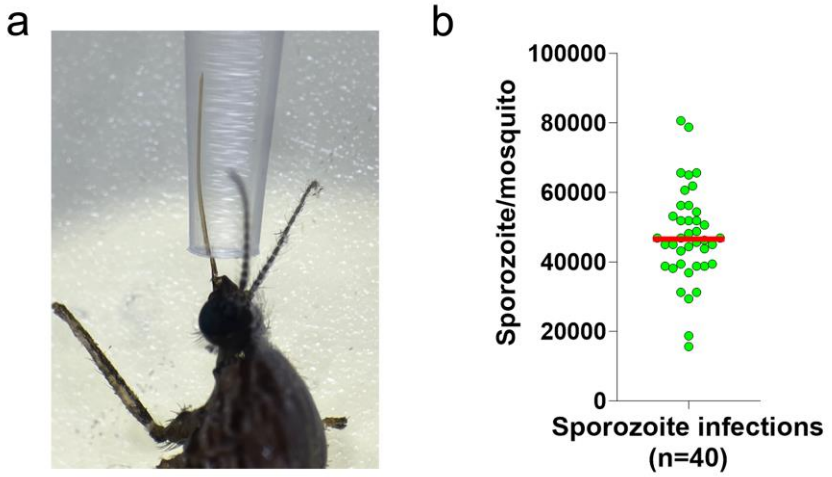

Supplementary Figure 10. a Collection of mosquito saliva. **b** Representative *P. berghei* sporozoite counting in infected salivary glands.

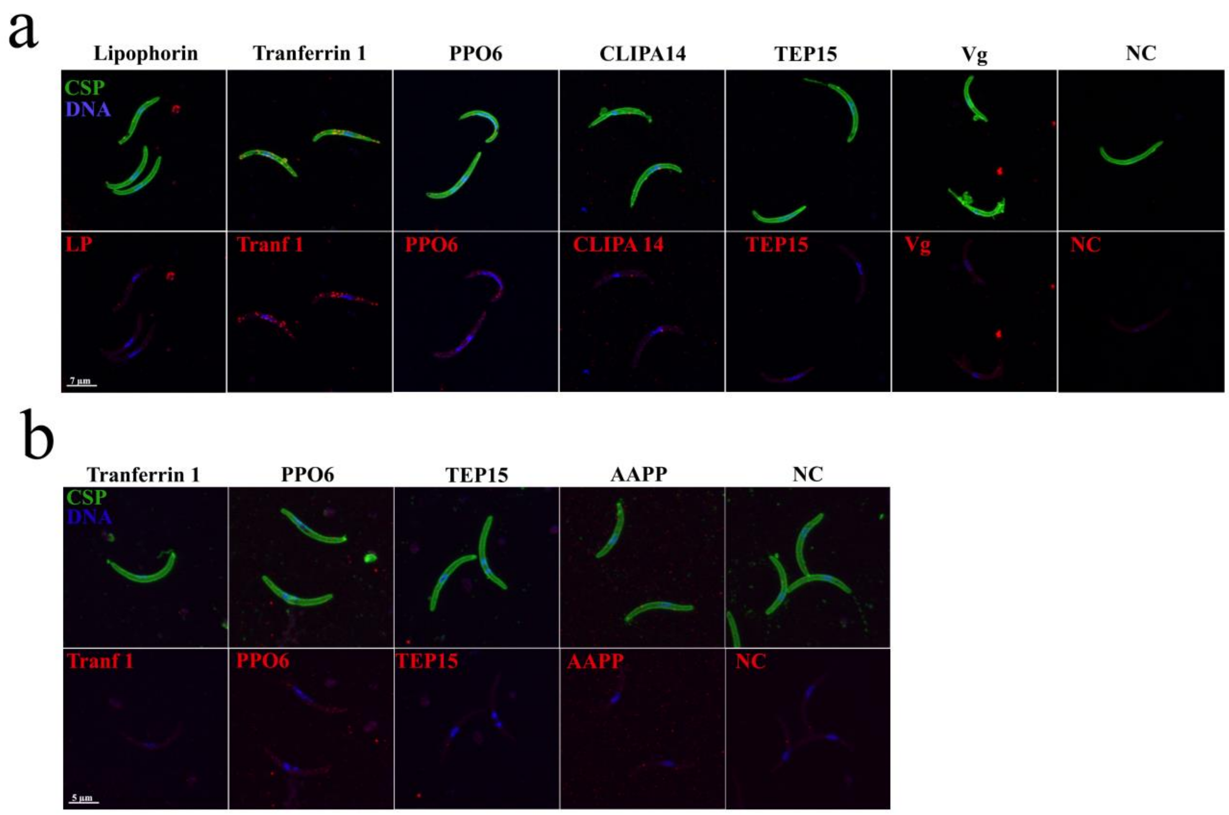

Supplementary Figure 11. Binding of hemolymph proteins to the surface of *P. berghei* sporozoites. **a** Sporozoites circulating in the mosquito hemolymph were collected by perfusion at 17 days post-infection. IFA labeling shows the circumsporozoite (CSP) protein in green and selected hemolymph proteins in red; primary antibodies were omitted in the negative control (NC). Confocal images

showing maximal projection. Scale bar: 7 µm. **b** Sporozoites were harvested from iSGs at 21 days post-infection. IFA labeling shows the circumsporozoite (CSP) protein in green and selected hemolymph proteins in red; primary antibodies were omitted in the negative control (NC). Confocal images showing maximal projection. Scale bar: 5 µm.

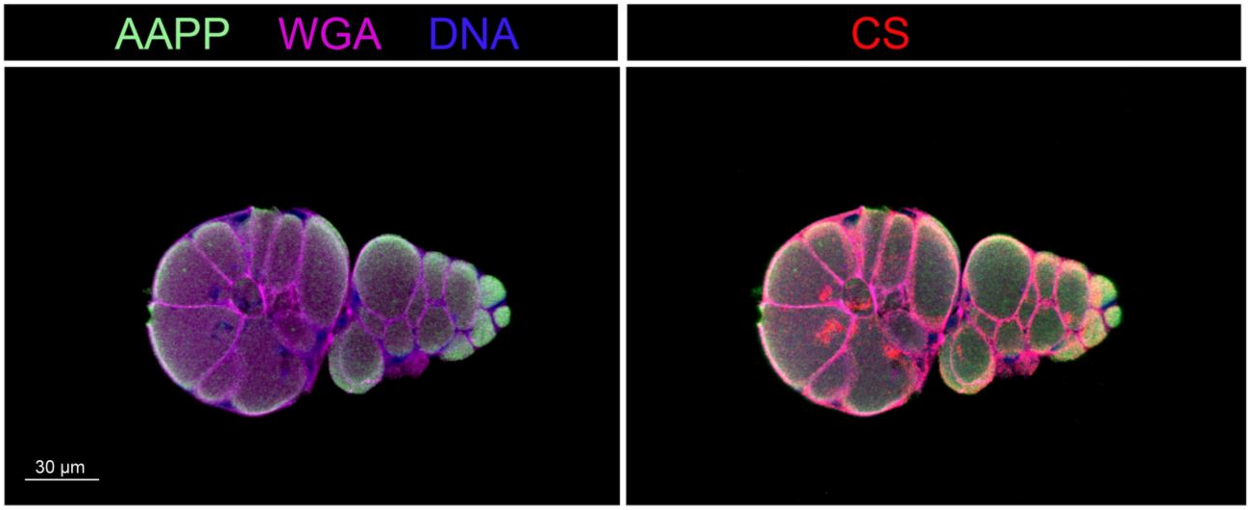

Supplementary Figure 12. Histological immunofluorescence of *P. berghei*-infected salivary glands. These images display the distribution of AAPP within the infected salivary gland. Staining includes AAPP (green), circunsporozoite (red), and WGA (magenta).

List of primers		
**A5R1**	qF	TCACCCAGTTCTTCGGCTAC
	qR	GGGCGTGCATCCAGATATTC
**Apyrase**	qF	TGCCATCGATCACTCCTTCA
	qR	CGATGCTCTGTACACGTTCG
**5'NTE**	qF	CGGGTACTGTCGATCGATGT
	qR	CATCCTTACCGGTGGCAATG
**D7R3**	qF	CGAACACCTTCTACACGTGC
	qR	GTTCCCATGTCCAGCTTTCC
**AAPP**	qF	CGAAAGGGAGCAGGAACTGT
	qR	CAAGATTCCAAGGGGCAACG
**Tranf1**	qF	ATCCGCATCGTGAACTCGAA
	qR	CCAGTGGCTGGAAGGTGAAA
**LP**	qF	CAGCCAGGATGGTGAGCTTAA
	qR	CACCAGCACCTTGGCGTT
**PPO6**	qF	CTGCCTAGAAGGGGCGATTT
	qR	CACATCCGGTTGGTCCAAGA
**CLIPA14**	qF	GGGTGCCATTGAACGTGTTT
	qR	CCGTTCGCGATAGCATTGTG
**S7**	qF	AGAACCAGCAGACCACCATC
	qR	GCTGCAAACTTCGGCTATTC

Supplementary Figure 13: Primer list.
