# Supplementary material for: High-resolution proteomics unveils salivary gland disruption and saliva-hemolymph protein exchange in Plasmodium-infected mosquitoes
Source: Nat Commun. 2025 Nov 20;16:10205. doi: 10.1038/s41467-025-64837-6 (PMC12635079; doi:10.1038/s41467-025-64837-6)
Supplement: Supplementary file 3 — Description of Additional Supplementary Files [file 41467_2025_64837_MOESM3_ESM.pdf]

Supplementary Data 1. List of salivary gland proteins from uninfected and *Plasmodium berghei*-infected *Anopheles gambiae* mosquitoes 21 days post-infection identified via LC-MS/MS-based label-free quantification (LFQ). Raw data were processed with MaxQuant and this table summarizes selected entries extracted from the proteinGroups.txt. LFQ Collum includes log_2-transformed, median-normalized LFQ intensities from MaxQuant across three biological replicates for each condition. Proteins were annotated using vector base and BLASTp against multiple databases.

Supplementary Data 2. *P. berghei* proteins identified by label-free quantification. Raw data were processed with MaxQuant and this table summarizes selected entries extracted from the proteinGroups.txt.

Supplementary Data 3. Protein abundance of different classes of proteins based on Gene Ontology. For abundance calculation, the relative iBaq for each class was calculated. Relative iBAQ values were calculated by summing the average of iBAQ intensities of proteins assigned to each functional class and normalizing by the total iBAQ across all quantified proteins in each sample. The last column includes the p-value from a two-sided t-test.

Supplementary Data 4. *Anopheles gambiae* proteins selected for differential expression. Proteins with at least four detections were used. The data is the same as that in Extended Data Table 1.

Supplementary Data 5. *Anopheles gambiae* proteins exclusively detected in uninfected samples with at least two quantified LFQs.

Supplementary Data 6. *Anopheles gambiae* proteins exclusively detected in *P. berghei*-infected samples with at least two quantified LFQs.

Supplementary Data 7. List of *Anopheles gambiae* proteins differentially expressed in the whole salivary gland proteome. Differential expression was determined with a two-sided, two-sample Student's t-test with permutation-based FDR control ($q \leq 0.05$) using a SAM-style variance parameter ($s_0 = 0.1$).

Supplementary Data 8. List of hemolymph proteins from uninfected and *Plasmodium berghei*-infected *Anopheles gambiae* mosquitoes 19 days post-infection identified via LC-MS/MS-based label-free quantification (LFQ). Raw data were processed using MaxQuant, and this table summarizes the selected entries extracted from the proteinGroups.txt file. LFQ Collum includes log2-transformed, median-normalized LFQ intensities from MaxQuant across two biological replicates for each condition. This list comprises proteins with at least three LFQ quantifications. Proteins were annotated using vector base and BLASTp against multiple databases. Differential expression was determined with a two-sided two-sample Student's t-test with permutation-based FDR control.

Supplementary Data 9. List of saliva proteins from uninfected and *P. berghei*-infected *Anopheles gambiae* mosquitoes 21 days post-infection identified via LC-MS/MS-based label-free quantification (LFQ). Raw data were processed using MaxQuant, and this table summarizes the selected entries extracted from the proteinGroups.txt file. LFQ Collum includes log2-transformed, median-normalized LFQ intensities from MaxQuant across four biological replicates for each

condition. Proteins were annotated using vector base and BLASTp against multiple databases.

Supplementary Data 10. List of saliva proteins from uninfected and *P. falciparum*-infected *Anopheles gambiae* mosquitoes 21 days post-infection identified via LC-MS/MS-based label-free quantification (LFQ). Raw data were processed using MaxQuant, and this table summarizes the selected entries extracted from the proteinGroups.txt file. LFQ Collum includes log2-transformed, median-normalized LFQ intensities from MaxQuant across four biological replicates for uninfected and three replicates for infected. Proteins were annotated using vector base and BLASTp against multiple databases.

Supplementary Data 11. Protein abundance of different classes of proteins based on Gene Ontology. For abundance calculation, the relative iBaq for each class was calculated. Relative iBAQ values were calculated by summing the average of iBAQ intensities of proteins assigned to each functional class and normalizing by the total iBAQ across all quantified proteins in each sample.

Supplementary Data 12. List of *Anopheles gambiae* proteins differentially expressed from the saliva proteome of uninfected and *P. berghei*-infected mosquitoes.

Supplementary Data 13. List of *Anopheles gambiae* proteins differentially expressed from the saliva proteome of uninfected and *P. falciparum*-infected mosquitoes. Differential expression was determined with a two-sided, two-sample Student's t-test with permutation-based FDR control ($q \leq 0.05$) using a SAM-style variance parameter ($s_0 = 0.1$).
