# Supplementary material for: High-resolution proteomics unveils salivary gland disruption and saliva-hemolymph protein exchange in Plasmodium-infected mosquitoes
Source: Nat Commun. 2025 Nov 20;16:10205. doi: 10.1038/s41467-025-64837-6 (PMC12635079; doi:10.1038/s41467-025-64837-6)
Supplement: Supplementary file 17 — Reporting Summary [file 41467_2025_64837_MOESM17_ESM.pdf]

nature portfolio

Corresponding author(s): Joel Vega-Rodriguez

Last updated by author(s): Sep 15, 2025

Reporting Summary

Nature Portfolio wishes to improve the reproducibility of the work that we publish. This form provides structure for consistency and transparency in reporting. For further information on Nature Portfolio policies, see our Editorial Policies and the Editorial Policy Checklist.

Statistics

For all statistical analyses, confirm that the following items are present in the figure legend, table legend, main text, or Methods section.

n/a	Confirmed	
☐	☒	The exact sample size (*n*) for each experimental group/condition, given as a discrete number and unit of measurement
☐	☒	A statement on whether measurements were taken from distinct samples or whether the same sample was measured repeatedly
☐	☒	The statistical test(s) used AND whether they are one- or two-sided<br>*Only common tests should be described solely by name; describe more complex techniques in the Methods section.*
☐	☒	A description of all covariates tested
☐	☒	A description of any assumptions or corrections, such as tests of normality and adjustment for multiple comparisons
☐	☒	A full description of the statistical parameters including central tendency (e.g. means) or other basic estimates (e.g. regression coefficient) AND variation (e.g. standard deviation) or associated estimates of uncertainty (e.g. confidence intervals)
☐	☒	For null hypothesis testing, the test statistic (e.g. *F*, *t*, *r*) with confidence intervals, effect sizes, degrees of freedom and *P* value noted<br>*Give P values as exact values whenever suitable.*
☒	☐	For Bayesian analysis, information on the choice of priors and Markov chain Monte Carlo settings
☒	☐	For hierarchical and complex designs, identification of the appropriate level for tests and full reporting of outcomes
☒	☐	Estimates of effect sizes (e.g. Cohen's *d*, Pearson's *r*), indicating how they were calculated

Our web collection on statistics for biologists contains articles on many of the points above.

Software and code

Policy information about availability of computer code

Data collection	Electron microscopy images were collected with a Tecnai T12 transmission electron microscope (Thermo Fisher) operating at 120 eV with a Rio digital camera (Gatan), a 70 kV Jeol JEM1400 TEM equipped with a TVIPS TemCam F416 4k x 4k pixel digital camera, or a Philips 410 Electron Microscope (Eindhoven, the Netherlands) under 80 kV<br>Spectophotometry data were collected on a Cytation 5 ® Cell Imaging Multi-Mode Reader (BioTek Instruments).<br>Histology images were collected on Leica ASP6025<br>Western blot data was collected on an iBright imager (ThermoFisher).<br>Confocal images were obtained using a Leica TCS SP8 DM8000 confocal microscope (Leica Microsystems, Wetzlar, Germany)<br>Quantitative PCR (qPCR) was done on a QuantStudio 7 real-time PCR system (Applied Biosystems)
Data analysis	Microscopy image processing was performed using Imaris 9.2.1 (Bitplane).<br>LC-MS/MS data were acquired using an Orbitrap Fusion Lumos mass spectrometer equipped with an EASY-Spray Ion Source and an EASY-nLC 1200 liquid chromatography system (Thermo Fisher Scientific)<br>LC-MS/MS data were processed using MaxQuant software (v2.0.3.0.)<br>Statistical analyses were done using Graphpad Prism version 9.0

For manuscripts utilizing custom algorithms or software that are central to the research but not yet described in published literature, software must be made available to editors and reviewers. We strongly encourage code deposition in a community repository (e.g. GitHub). See the Nature Portfolio guidelines for submitting code & software for further information.

Data

Policy information about availability of data

All manuscripts must include a data availability statement. This statement should provide the following information, where applicable:

- Accession codes, unique identifiers, or web links for publicly available datasets
- A description of any restrictions on data availability
- For clinical datasets or third party data, please ensure that the statement adheres to our policy

The datasets generated during and/or analysed during the current study are available from the corresponding author on reasonable request.

Research involving human participants, their data, or biological material

Policy information about studies with human participants or human data. See also policy information about sex, gender (identity/presentation), and sexual orientation and race, ethnicity and racism.

Reporting on sex and gender	NA
Reporting on race, ethnicity, or other socially relevant groupings	NA
Population characteristics	NA
Recruitment	NA
Ethics oversight	NA

Note that full information on the approval of the study protocol must also be provided in the manuscript.

Field-specific reporting

Please select the one below that is the best fit for your research. If you are not sure, read the appropriate sections before making your selection.

[x] Life sciences [ ] Behavioural & social sciences [ ] Ecological, evolutionary & environmental sciences

For a reference copy of the document with all sections, see nature.com/documents/nr-reporting-summary-flat.pdf

Life sciences study design

All studies must disclose on these points even when the disclosure is negative.

Sample size	No statistical methods were used to determine sample size. Sample size was determined to be adequate based on experimental consistency and effect, and previous publications. Generally three independent biological replicates were done for each experiment.
Data exclusions	No data were excluded from analysis
Replication	All replicates were successful and included in data analysis
Randomization	Randomization was not necessary for our experiments.
Blinding	Blinding wasn't necessary

Reporting for specific materials, systems and methods

We require information from authors about some types of materials, experimental systems and methods used in many studies. Here, indicate whether each material, system or method listed is relevant to your study. If you are not sure if a list item applies to your research, read the appropriate section before selecting a response.

Materials & experimental systems

n/a	Involved in the study
	[x] Antibodies
[x]	Eukaryotic cell lines
[x]	Palaeontology and archaeology
	[x] Animals and other organisms
[x]	Clinical data
[x]	Dual use research of concern
[x]	Plants

Methods

n/a	Involved in the study
[x]	ChIP-seq
[x]	Flow cytometry
[x]	MRI-based neuroimaging

Antibodies

Antibodies used

anti-TEP15 rabbit monospecific IgG(1 µg/ml, Pacific Immunology), anti-Transferrin 1 rabbit monospecific IgG (1 µg/ml, Pacific Immunology), anti-PPO6 rabbit polyclonal serum (1:500) — kindly provided by Dr. Ryan Smith from Iowa State University (Kwon and Smith, 2019, PMID: 31235594) —, anti-AAPP rabbit monospecific IgG (1 µg/ml, Pacific Immunology), anti-Lipophorin monospecific IgG (1 µg/ml, Pacific Immunology), anti-CLIPA14 (1:400, Boster Bio), and anti-circumsporozoite (CS) protein mouse monoclonal antibody (3D11, monospecific IgG). Alexa Fluor 488-conjugated, Alexa Fluor 594-conjugated goat anti-mouse or goat anti-rabbit antibodies (1:1000, Thermo Fisher), Wheat Germ Agglutinin (WGA) conjugated to Alexa Fluor 647 (Thermo Fisher) at 2 µg/ml. Slides were counterstained with Hoechst 33342 (20 µM, Thermo Fisher)

Validation

anti-TEP15 rabbit monospecific IgG(1 µg/ml, Pacific Immunology), anti-Transferrin 1 rabbit monospecific IgG (1 µg/ml, Pacific Immunology), anti-PPO6 rabbit polyclonal serum (1:500) — kindly provided by Dr. Ryan Smith from Iowa State University (Kwon and Smith, 2019, PMID: 31235594) —, anti-AAPP rabbit monospecific IgG (1 µg/ml, Pacific Immunology), anti-Lipophorin monospecific IgG (1 µg/ml, Pacific Immunology), anti-CLIPA14 (1:400, Boster Bio), and anti-circumsporozoite (CS) protein mouse monoclonal antibody (3D11, monospecific IgG). Alexa Fluor 488-conjugated, Alexa Fluor 594-conjugated goat anti-mouse or goat anti-rabbit antibodies (1:1000, Thermo Fisher), Wheat Germ Agglutinin (WGA) conjugated to Alexa Fluor 647 (Thermo Fisher) at 2 µg/ml. Slides were counterstained with Hoechst 33342 (20 µM, Thermo Fisher)

Animals and other research organisms

Policy information about studies involving animals; ARRIVE guidelines recommended for reporting animal research, and Sex and Gender in Research

Laboratory animals

Anopheles gambiae Keele strain (Hurd et al., 2005), Female, 3-21 days old
BALB/c mice, Female, 4-6 weeks old, NIAID animal study proposals LMVR-22

Wild animals

The study did not involve wild animals

Reporting on sex

NA

Field-collected samples

The study did not involve wild animals

Ethics oversight

Studies with animals were approved by the Institutional Animal Care and Use Committee (IACUC), Care and Use Committees (NIAID ACUC)
Swiss webster mice, Female, 4-6 weeks old, NIAID animal study proposals LMVR-22

Note that full information on the approval of the study protocol must also be provided in the manuscript.
